# Reply to Kodner et al.: Fundamental misunderstanding of both model and methods

**DOI:** 10.1073/pnas.2204944119

**Published:** 2022-07-05

**Authors:** Steven T. Piantadosi, Yuan Yang

**Affiliations:** ^a^Department of Psychology, Helen Wills Neuroscience Institute, University of California, Berkeley, CA 94720;; ^b^College of Computing, Georgia Institute of Technology, Atlanta, GA 30332

Our recent work (1) shows a program-learning model can acquire some key structures in natural language, including recursive hierarchies and patterns that require more than context-free capacities (2).

Kodner et al.’s (KCY) commentary (3) is based on several fundamental misunderstandings. Most notably, they claim that our “model transforms candidate hypotheses into probabilistic context-free grammars that are evaluated against the training data via Bayesian inference.” This is unambiguously incorrect: At no point does our model convert hypotheses into probabilistic context-free grammars. Not only does it not do that, but that approach could not work because we show that the model can learn languages that are not context-free: No method of comparing context-free grammars could learn the languages our model succeeds on. Our model compares programs, not grammars, and we showed examples of these programs in the paper.

KCY claim we conceive of “language as strings” and we fail to recognize that language has structure. This is another fundamental misunderstanding. It is true that the data provided to the model are strings (sequences of characters), following prior work on learnability, but the model uses the strings to discover structure, much like a linguist would. Finding latent structure behind strings is the only way for the model to generalize beyond what it has seen, which our results document it does.

**Fig. 1.**
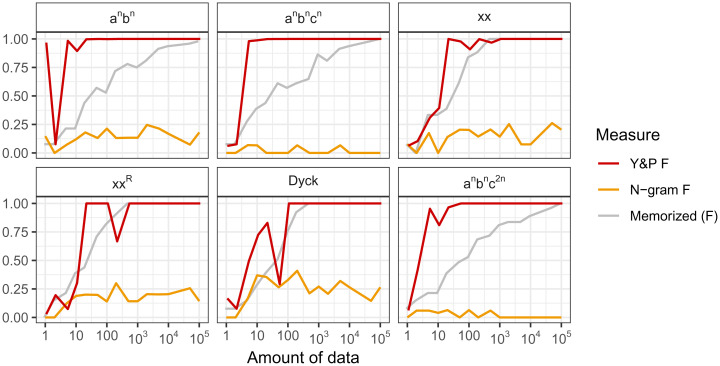
F-scores (*y* axis) as a function of amount of data (*x* axis) for Yang and Piantadosi’s (1) learning model (red), a learner who memorizes data (gray), and the n-gram model suggested by Kodner et al. (3). Our F-score measure works well: It correctly shows that an n-gram model can learn finite-state languages (figure 1 in ref. 3) and here correctly shows an n-gram model cannot learn these non-finite-state languages.

KCY contend that our analysis method is flawed because an n-gram model can show high performance on some languages when following our methods. Their interpretation is not correct. KCY only examined performance of an n-gram model on finite-state languages (figure 1 of ref. 3), which are precisely languages that an n-gram model can represent. So, of course an n-gram model can do well on their examples. In fact, no evaluation metric could show that n-gram models are poor on these languages because, simply, they are not. One has to look at nonfinite state languages, where our evaluation scheme shows an n-gram model fails (Fig. 1). Thus, our evaluation does exactly what it should: It scores an n-gram model high on languages they can learn (figure 1 of ref. 3) and low on languages they cannot learn (Fig. 1). The same metric shows that our model learns everything from finite sets to context-sensitive grammars.

Finally, KCY grab onto our statement that people do not necessarily use the same methods as our implementation. Ours is a standard Marr (4) computational-level analysis: We hoped to formalize the problem people solve (Bayesian selection of generative processes) without necessarily knowing how they solve it. Our more modest claim is warranted because there is no evidence about how children solve this problem. The insight of Marr also answers KYC’s final question of how the model helps us understand acquisition. The model shows that learning a generating process for languages is possible, contradicting standard claims in the field, including a generative syntax textbook we cite. Our best guess for how the field got it wrong for so long is epitomized by KCY’s commentary: Some researchers have been so eager to dismiss learning, they have done so without understanding how it works.
